# Effect of the Chemical Structure of Ionic Glycolipids on Their Lyotropic Aqueous Phase Behavior

**DOI:** 10.1021/acsomega.5c08922

**Published:** 2025-11-07

**Authors:** Giuliana Valentini, Tomás S. Plivelic, Paulo R. A. F. Garcia, Shinji Kihara, Ben J. Boyd, Watson Loh

**Affiliations:** † Institute of Chemistry, State University of Campinas (UNICAMP), P.O. Box 6154, 13083-970 Campinas, Brazil; ‡ Department of Pharmacy, Faculty of Health and Medical Sciences, 4321 University of Copenhagen, Copenhagen 2100, Denmark; § MAX IV Laboratory, Lund University, 224 84 Lund, Sweden; ∥ Drug Delivery, Disposition and Dynamics, Monash Institute of Pharmaceutical Sciences, 2541 Monash University, Parkville, VIC 3052, Australia

## Abstract

This study examines the self-assembly and lyotropic aqueous phase behavior of galactose- (GC) and rhamnose-based (RC) bioinspired glycolipids with C_10_ and C_14_ alkyl chains using small-angle X-ray scattering and wide-angle X-ray scattering measurements. In the isotropic micellar regime (L_1_), ellipsoidal micelles were identified, with their size and aggregation number mainly controlled by the surfactant alkyl chain length. As the concentration increases, both GC and RC systems form hexagonal (H_1_) mesophases, with the C_14_ homologues exhibiting a broader stability range of H_1_ and crystallizing above 80 wt %. Notably, above 95 wt % RC10 assembles into an additional bicontinuous cubic Ia3d phase, which was not observed in the GC systems. Phase boundary diagrams further indicate that the presence of rhamnose, with fewer hydroxyl groups and weaker hydrogen-bonding ability, favors mesophase ordering at lower concentrations, while galactose enhances hydration and expands the L_1_ domain. At higher glycolipid concentrations, however, headgroup interactions may alter this trend, and the hydroxyl-rich galactose tends to promote crystallization, while rhamnose maintains the H_1_ mesophase up to a more concentrated region. Overall, these findings demonstrate that bioinspired glycolipid self-assembly depends on a balance of contributions from their alkyl chains and sugar headgroups, transitioning from a sugar solubility-driven mechanism in L_1_ to prevailing sugar–sugar interactions in the concentrated regime.

## Graphical abstract

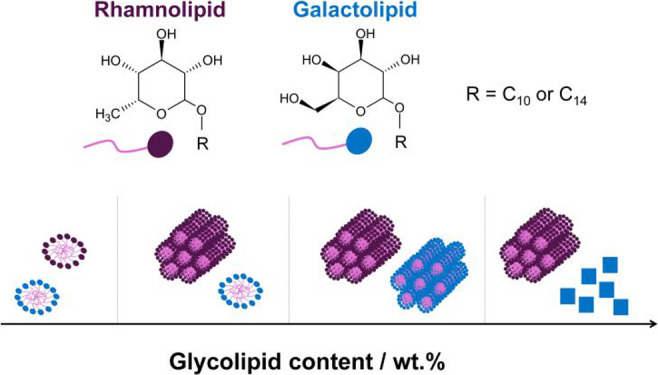

## 1 Introduction

Glycolipids are amphiphilic molecules composed of a hydrophobic alkyl chain and a hydrophilic sugar headgroup.^1–4^ A fundamental understanding of how features such as alkyl chain length, headgroup structure, and temperature influence the self-assembly of glycolipids is crucial for tailoring their physicochemical properties.^5^ The formation of lyotropic liquid crystalline mesophases is susceptible to these molecular parameters, as they directly impact the molecular packing and their critical packing parameters (CPP), which govern the preferred curvature of the aggregates.^6,7^ Extensive studies have been conducted on nonionic sugar-based surfactants, such as glucose and maltose derivatives.^8–13^ However, the lyotropic behavior of ionic glycolipids, and specifically how headgroup identity and hydrophobic chain length jointly determine mesophase formation, has received far less attention and remains poorly understood.

Among the various glycolipids, rhamnolipids, and galactolipids have attracted significant attention due to their biocompatibility, biodegradability, and versatile self-assembly properties.^10,200^ Rhamnolipids, which display rhamnose as their sugar headgroup (Scheme 1), are widely studied for applications in biotechnology and environmental remediation.^14–17^ In contrast, galactolipids, featuring a galactose headgroup, are naturally abundant in plant membranes and play important roles in biological recognition and membrane dynamics.^200^ Despite their relevance, a direct and systematic comparison of rhamnolipids and galactolipids that isolates the effects of sugar headgroup and alkyl chain length on self-assembly has not yet been reported.

**Scheme 1:**
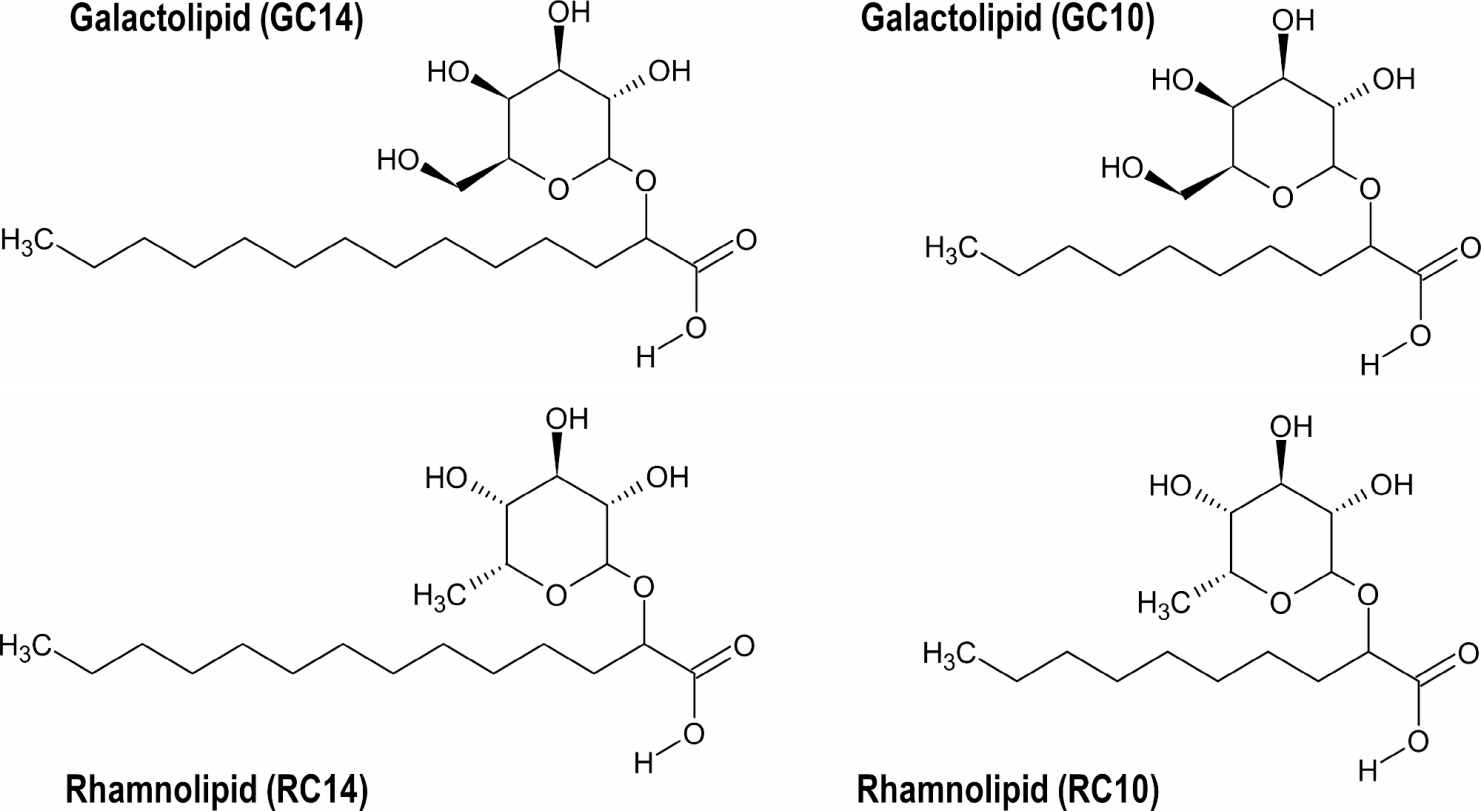
Chemical Structures of the Glycolipids Used: Galactolipid C_14_ (GC14), Galactolipid C_10_ (GC10), Rhamnolipid C_14_ (RC14), and Rhamnolipid C_10_ (RC10)

Previous research has demonstrated that changes in molecular architecture, such as increasing the hydrophobic chain length from C_10_ to C_14_ or modifying the sugar headgroup from rhamnose to galactose, can drastically affect the aggregation behavior and the resulting mesophase structures.^2,18^ However, the extent to which these molecular variations influence the self-assembly of ionic glycolipids remains poorly understood, particularly in aqueous environments where electrostatic interactions and headgroup hydration play a crucial role.^19^ One key issue is the limited availability of these glycolipids in high enough purity, especially when they are extracted from natural products or fermentation broths, which limits building studies devoted to elucidating their structure–property relationships.

In this study, the aqueous phase behavior of four bioinspired synthetic glycolipids, differing in their sugar headgroups (rhamnose versus galactose) and alkyl chain lengths (C_10_ versus C_14_), as illustrated in Scheme 1, was systematically investigated. Binary mixtures with water at varying compositions were prepared, up to the highest concentrations possible to handle, investigating the impact of their molecular structure on self-assembly. We recently reported an initial study on their micellization and surface activity,^20^ which indicated significant differences among the glycolipid families, likely due to variations in interactions involving their sugar headgroups. In the present study, we extend that investigation toward concentrations at which they form mesophases, mapping their whole lyotropic phase behavior.

To solve their nanoscale structural organization and to identify the mesophases formed, Small- and Wide-Angle X-ray Scattering (SAXS/WAXS) measurements were employed. These techniques provide structural information, allowing for precise characterization of periodicity associated with different liquid crystalline phases. By correlating their structural parameters with their molecular architecture, this work aims to advance the understanding of how ionic glycolipids self-assemble into ordered nanostructures. The insights obtained here are expected to contribute to the rational design of sugar-based amphiphiles for different potential applications.

## 2 Materials and Methods

The commercial glycolipids galactolipid C_14_ (GC14), galactolipid C_10_ (GC10), rhamnolipid C_14_ (RC14), and rhamnolipid C_10_ (RC10) used in this study were purchased from Glycosurf (Salt Lake City, USA). All samples had a purity greater than >95% and were used without further treatment. The water used in the experiments was of Milli-Q Plus grade.

### 2.1 Determination of Phase Boundaries

To obtain information on their binary phase diagrams, samples containing glycolipids C_10_ or C_14_ and water were prepared at varying concentrations (weight percent, wt %) to evaluate the phases formed. These samples were analyzed in a constant-temperature environment, thereby producing phase boundaries that were used to elucidate their lyotropic phase behavior. The samples containing RC10, GC10, and RC14 were incubated at 25 °C. The GC14 experiments were conducted at 63 °C, which is above its Krafft temperature (see Section 1 in the Supporting Information). While experiments were conducted in pure water to probe the intrinsic behavior of the glycolipids, it is acknowledged that ionization and phase boundaries may differ under buffered or physiological conditions. Nevertheless, as the glycolipids display the same p*K*_a_ values, their ionization state should be the same and, therefore, the observed qualitative trends and structure–function relationships are expected to remain valid.

Mixtures with appropriate amounts of glycolipid and water were placed in glass tubes, then sealed and subjected to centrifugation in at least 8 cycles of 60 min each, at 5000 rpm, once a day, over 7 days. After each cycle, the glass tube was turned upside down, and a new centrifugation was performed under the same conditions. Final centrifugation was made with the upright tube, which was then analyzed for homogeneity. The samples were stored for 30 days at the selected temperature (25 °C for GC10, RC14, and RC10) before the measurements. GC14 samples were maintained at 63 °C for at least 7 days before measurement, with no visible yellowing, ruling out the occurrence of significant degradation.

### 2.2 Small-Wide Angle X-ray Scattering (SAXS-WAXS) Measurements

SAXS/WAXS measurements were conducted at the CoSAXS beamline of the MAX IV Laboratory synchrotron (Lund, Sweden)^21^ to identify the mesophases in the systems as a function of their composition. SAXS measurements were performed using a sample-to-detector distance of 1.5 m and a monochromatic X-ray wavelength of 1.0 Å. SAXS data were collected using an Eiger2 4 M hybrid pixel X-ray detector. The system was calibrated using silver behenate (AgBe), covering a *q*-range of approximately 0.1–5.0 nm^–1^, where *q* = (4π/λ) sin­(θ) and 2θ is the scattering angle. WAXS data were simultaneously recorded using an L-shaped Pilatus3 2 M detector, positioned at a sample to detector distance of 0.5 m. The WAXS detector covered the *q*-range of 1.0–20 nm^–1^, enabling further analyses of the molecular organization and short-range order.

An exposure time of 0.05 s per frame was used, with 10 exposures taken along the vertical direction of the capillary. Data reduction, including background subtraction and azimuthal integration, was performed using beamline Jupyther notes. The dimensions of the ordered self-assembled structures were calculated from the peak positions in the SAXS profiles using the following relationships.^22^

For a two-dimensional hexagonal (H_1_) structure, the unit cell lattice parameter *a* was obtained from eq 1.

$$d_{hk}=\frac{\sqrt{3a}}{2\sqrt{\left(h^{2}+hk+k^{2}\right)}}$$ 1

where *d_hk_* is the interplanar distance associated with the Bragg peak with Miller indices (*hk*) and *d_hk_* = 2π/*q*_*hk*_.

In a plot *d_hk_* vs $1/\sqrt{\left(h^{2}+hk+k^{2}\right)}$ , *a* is obtained as a linear fitting parameter using the angular coefficient *m* present in eq 2:

$$a=\frac{2m}{\sqrt{3}}$$ 2

The cell lattice parameter (*a_hkl_*) of the cubic gyroid phase Ia3d structure was calculated from the Miller indices of the reflections for the cubic phase described by eq 3:

$$a_{hkl}=\frac{2\pi }{q_{i}}\sqrt{\left(h^{2}+k^{2}+l^{2}\right)}$$ 3

### 2.3 SAXS Model Used to Analyze Data from the Micellar Phase (L_1_)

For the micellar phase (L_1_), the sample scattering intensity was described by a core–shell ellipsoid of revolution.^23,24^ This model has already been applied to investigate aggregates formed by some of these glycolipids at concentrations around their critical micelle concentrations.^20^

The main equation of this model is

$$I(q)=A\cdot P_{\mathrm{CS}-\mathrm{Ell}}(q,\rho ,R,\epsilon ,t_{h})+B$$ 4

where *A* is the scale factor, ρ is the electron density contrast between the core and the shell, *R* is the radius of the ellipsoid core, ϵ is the eccentricity, *t*_*h*_ is the thickness of the shell, and *B* is a constant describing the background scattering. The form factor for the core–shell ellipsoid of revolution, *P*_CS_, is given by

$$P_{\mathrm{CS}}(q,\rho ,R,\epsilon ,t_{h})=\int_{0}^{\pi /2}F_{\mathrm{CS}}^{2}(q,\rho ,R,\epsilon ,\alpha ,t_{h})\sin\alpha d\alpha$$ 5

$$F_{\mathrm{CS}}^{2}(q,\rho ,R,\epsilon ,\alpha ,t_{h})=\{V_{\mathrm{out}}F_{\mathrm{sph}}[q,r(R,\epsilon ,\alpha )]-(1-\rho )V_{\mathrm{in}}F_{\mathrm{sph}}[q,r(R+t_{h},\epsilon ,\alpha )]{\}}^{2}$$ 6

$$F_{\mathrm{sph}}[q,r(R,\epsilon ,\alpha )]=3\frac{\sin[qr(R,\epsilon ,\alpha )]-qr(R,\epsilon ,\alpha )\cos[qr(R,\epsilon ,\alpha )]}{[qr(R,\epsilon ,\alpha ){]}^{3}}$$ 7

The orientation of the particle relative to the scattering vector was given by the angle α, which was integrated over π/2 to account for randomly oriented particles in solution. The effective radius of the ellipsoid along a given orientation was expressed as *r*(*R*,ϵ,α), which was used in the expression for the spherical amplitude *F*_sph_. Finally, the volumes *V*_out_ and *V*_in_ were the volumes of the shell and core ellipsoids, respectively.^25^ To account for interparticle interactions in the system, a hard-sphere structure factor based on the analytical solution of the Percus–Yevick potential (*S*_PY_(*q*, *R*_HS_)) was used.^24,26^ The effective hard-sphere radius, *R*_HS_, was approximated as the outer radius of the ellipsoids averaged over all orientations. The structure factor is described in eq 8:

$$S_{\mathrm{PY}}(q,R_{\mathrm{HS}})=\frac{1}{1+24\eta G(2R_{\mathrm{HS}}q)/(2R_{\mathrm{HS}}q)}$$ 8

where *R*_HS_ is the effective hard-sphere radius, η is the volume fraction of hard spheres, and *G* is defined in Section 2 in the Supporting Information. The entire data fitting process was performed using custom software developed in C++.

## 3 Results and Discussion

### 3.1 Distribution of Glycolipid Species as a Function of Their Concentration

The glycolipids used in this study are derived from fatty acids, containing carboxylic groups and, therefore, behave as weak acids. The p*K*_a_ values for galactose (GC) and rhamnose (RC) glycolipids were determined in an earlier study^20^ as 4.1 ± 0.1 and 4.3 ± 0.3, respectively. Consequently, at pH values close to their p*K*_a_, the ratio between ionized and nonionized species will vary according to the Henderson–Hasselbach equation:

$$pH-pK_{a}=\log\left[\frac{{\mathrm{COO}}^{-}}{\mathrm{COOH}}\right]$$ 9

The p*K*_a_ values were determined in the micellar region, above the CMC and, therefore, describe the apparent ionization behavior of glycolipids in their aggregated state rather than as isolated monomers. Under these conditions, the measured pH values were approximately 5.0 ± 0.5 for 10–15 wt % glycolipid solutions, indicating partial ionization of the carboxylic headgroups within a more acidic micellar microenvironment compared to the bulk phase. In the analyses presented below, the mixtures are treated as binary (glycolipid + water) systems. However, it should be noted that the proportion of glycolipid molecules may vary, which could potentially influence their aggregation behavior. One clear indication of such an effect is the comparison of the Krafft temperature determined for the GC14 glycolipid in water (see Section 1 in the Supporting Information), which is well above that determined in buffer solutions at pH 7.4,^20^ in which the glycolipids were fully ionized and, consequently, much more soluble in water.

This condition is less controlled but more representative of practical environments, reflecting the most likely scenario in which these and similar glycolipids would be used, namely in aqueous rather than buffered solutions. Moreover, at the high concentrations investigated, buffer levels would need to be so high that additional effects, such as ion–ion interactions arising from high ionic strength, would have to be considered. Therefore, we believe that the present data on their aqueous phase behavior are more relevant for practical purposes, while still allowing meaningful comparisons based on their chemical structures, as discussed below.

### 3.2 Concentrated Glycolipid Micellar Phases

In an earlier study, we investigated the micelles formed in buffer pH 7.4 by some glycolipids used in the present study,^20^ above their critical micelle concentrations, along with other properties of these glycolipids. SAXS analyses revealed that their micelles are slightly elongated, with dimensions that varied with their sugar and alkyl groups.

In the present study, their L_1_ phases were analyzed at higher concentrations to investigate potential differences in their micellar structures. The SAXS results are presented in Figure 1, and their most important micellar parameters are summarized in Table 1.

**Figure 1:**
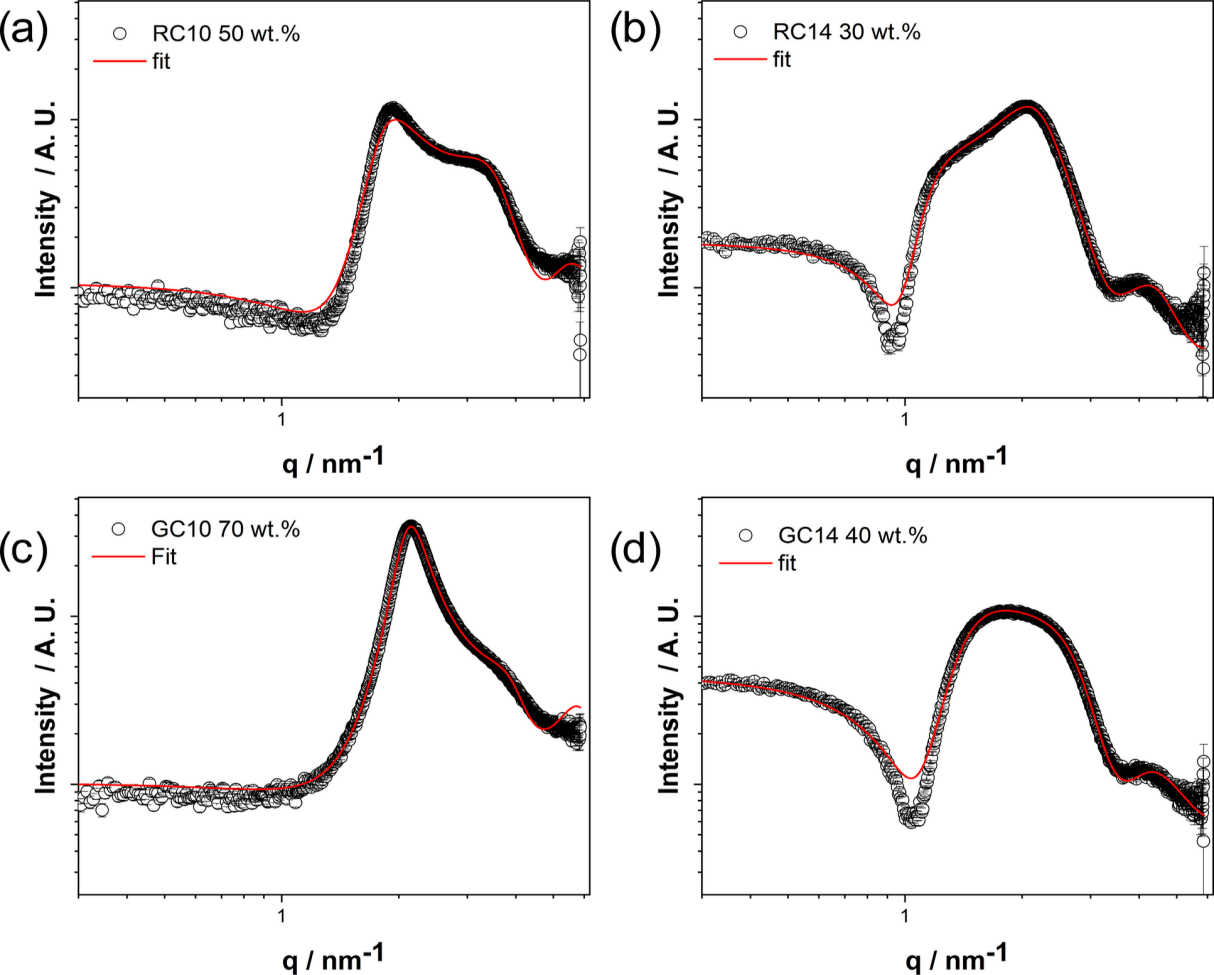
SAXS curves of the micellar phases for RC10 50 wt % (a), RC14 30 wt % (b), GC10 70 wt % (c), and GC14 40 wt % (d)—red lines represent best fitting. For RC10, RC14, and GC10, the measurements were conducted at 25 °C, while for GC14, they were performed at 63 °C.

**Table 1:** Micellar Parameters Obtained from Fitting of the SAXS Profiles

	RC10 50%	RC14 30%	GC10 70%	GC14 40%
*R*/nm	0.978 ± 0.001	1.284 ± 0.003	1.112 ± 0.002	1.209 ± 0.001
*t_h_*/nm	0.465 ± 0.004	0.582 ± 0.002	0.265 ± 0.001	0.639 ± 0.01
ϵ	1.618 ± 0.001	1.510 ± 0.004	1.535 ± 0.001	1.518 ± 0.001
*V*_tail_/nm^3^	0.269	0.377	0.269	0.377
*V*_micelle core_/nm^3^	6.34 ± 0.02	13.4 ± 0.2	8.83 ± 0.04	11.2 ± 0.01
*N*_app core_	14 ± 1	28 ± 1	33 ± 1	30 ± 1
*V*_micelle core+shell_/nm^3^	11.5 ± 0.2	36.7 ± 0.2	15.8 ± 0.1	35.4 ± 0.5
*N*_app core+shell_	43 ± 1	97 ± 1	59 ± 1	94 ± 1

The values were obtained using a core–shell ellipsoid model combined with a hard-sphere structure factor (eqs 4–8). Core radius (*R*), shell thickness (*t*_*h*_), and eccentricity (ϵ). *V*_tail_ corresponds to the volume occupied by a single hydrophobic tail (C_10_ or C_14_), calculated using approximations.^27,28^ *V*_micelle core_ and *N*_agg core_ represent the core volume and aggregation number, respectively, calculated from the fitting parameters, excluding the shell. V_micelle core+shell_ and *N*_agg core+shell_ correspond to the total volume and aggregation number, including the shell thickness. For details about the volume calculation, see Section 3 in the Supporting Information.

The fitting model was based on the form factor of ellipsoids combined with the hard-sphere structure factor, as previously described.^29–31^ In particular, the ellipsoidal core–shell form factor was employed to capture the scattering features more accurately. As noted earlier, the depth of the form factor minimum (Figure 1) around *q* ≈ 1.0 nm^–1^ is well described by a core–shell structure. The SAXS data were well described by an ellipsoidal micelle model. Although micellar polydispersity can influence aggregate shape,^10^ the polydispersity parameter was not significant in our fits, and the observed morphology remains consistent with trends reported for related glycolipids near the sphere-to-ellipsoid transition. The ellipsoidal model was subsequently applied to fit the data for RC10 50 wt %, RC14 30 wt %, GC10 70 wt %, and GC14 40 wt %, all measured before the formation of their hexagonal (H_1_) phases. The form factor parameters obtained from the fittings are listed in Table 1.

In the RC and water systems, the core radius (*R*) ranges from 0.978 ± 0.001 nm (RC10 50%) to 1.284 ± 0.003 nm (RC14 30%), indicating that micelles formed by glycolipids with longer alkyl chains (C_14_) display a larger core, as expected due to the increase in their hydrophobic chain. The same trend was observed for both GC systems, with a radius of 1.112 ± 0.002 nm for GC10 70 wt % and of 1.209 ± 0.001 nm for GC14 40 wt %. These *R* values are consistent with those reported in their dilute solutions in phosphate buffer systems, where a similar model was applied,^20^ yielding values of 1.68 ± 0.01 nm for RC14 and 1.55 ± 0.01 nm for GC14. Interestingly, the increased ionization of these glycolipids at pH 7.4 does not significantly affect their aggregate dimensions. The similarity between the micellar dimensions of GC14 in the previous study and those obtained for the other systems in this work indicates that, despite being measured at a different temperature, the micellar structure is only minimally affected.

The shell thickness (*t*_*h*_) also varies among the systems, with lower values for GC10 70% (0.265 ± 0.001 nm) and higher values for GC14 40% (0.639 ± 0.01 nm), indicating differences in the arrangement and packing of the hydrophilic headgroups at the micelle interface. This variation may also reflect differences in the interaction of the polar headgroups with the aqueous environment or the nature of their functional groups. The eccentricity (ϵ), which describes the distortion of the ellipsoid relative to a sphere, shows relatively consistent values between 1.510 ± 0.004 and 1.618 ± 0.001. Comparing all the glycolipids, the micelles formed by the C_10_ appear more distorted, which may be explained by their shorter hydrophobic tail and the difficulty in packing into a perfect sphere.^7^

Based on the micelle fitting parameters, two approaches were considered: using only the core dimensions or including the core plus shell thickness to better relate micelle size to glycolipid structure. Each approach has limitations: the core-only model underestimates size by excluding the sugar headgroup, while including the shell overestimates due to water content. The “true aggregation number” likely lies between these estimates, and both are discussed in this work.

The micelle including the shell (*V*_micelle core+shell_) followed the expected trend, presenting larger values for the C_14_-based systems compared to the C_10_-based systems, directly related to the larger size of the longer hydrophobic chains. The difference between the core and core+shell volumes highlights the significant contribution of the shell to the overall micellar structure. To provide a consistent analysis, the aggregation number (*N*_app core+shell_) was calculated using the core+shell model. This approach accounts for the entire micellar structure, including the hydrated shell region, which is expected to contribute to the effective micelle volume, and provides a better representation of the number of glycolipid molecules per micelle. The calculated (*N*_app core+shell_) values display the expected trend, with larger aggregation numbers for RC14 30 wt %, indicating that micelles formed from longer alkyl chain bioinspired glycolipids are larger.

In contrast, using only the core volume (ignoring the shell) systematically underestimates these values (*N*_app core_) relative to ones experimentally estimated from other techniques. For example, calculations assuming a spherical hydrophobic core yield aggregation numbers of 40 (C_10_) and 73 (C_14_),^27,28^ which are lower than the values obtained when the shell is included. Therefore, the core+shell model provides a more realistic estimation for these systems and was the preferred approach here. Additionally, at these concentrations, there was no evidence for the formation of more elongated or rod-like micelles.

Due to the high concentrations of these systems, the hard-sphere structure factor (*R*_HS_) was determined for each system (see Section 3 of the Supporting Information). The R_HS_ parameter, which reflects the interaction distance between micelles, was obtained for RC14 30% (2.827 ± 0.003 nm), GC14 (2.209 ± 0.005 nm), RC10 (1.762 ± 0.002 nm), and GC10 (1.591 ± 0.001 nm). Because the concentration differs for each curve, these values cannot be directly compared; however, they are consistent with the micelle dimensions for C_14_ and C_10_ indicated by their radii (*R*) in Table 1. The good fit obtained with the hard-sphere model suggests that the effective micellar charge is very low, meaning that electrostatic interactions contribute little to micelle organization under the studied conditions. As the glycolipid concentration increases, counterion screening becomes more pronounced, leading to a further reduction in effective surface charge and a decrease in electrostatic repulsion between micelles, which in turn influences their packing and phase behavior.

Comparing the micelles in the dilute aqueous regime with those previously reported in phosphate buffer,^20^ no significant changes in the micelle aspect ratio were observed with increasing concentration. SAXS analyses showed no features indicating elongated or rod-like aggregates, even at concentrations very close to the H_1_ phase boundary, suggesting that micelles remain predominantly ellipsoidal under the conditions studied.

### 3.3 Lyotropic Phase Behavior of Rhamnolipids RC10 and RC14

To investigate the relationship between the lyotropic phase behavior of ionic glycolipids and their molecular features, specifically the effects of their sugar headgroups and hydrophobic chains, SAXS/WAXS measurements were performed on their aqueous mixtures. Figure 2 presents the SAXS and WAXS curves for different rhamnolipids RC14 and RC10 as a function of their composition.

**Figure 2:**
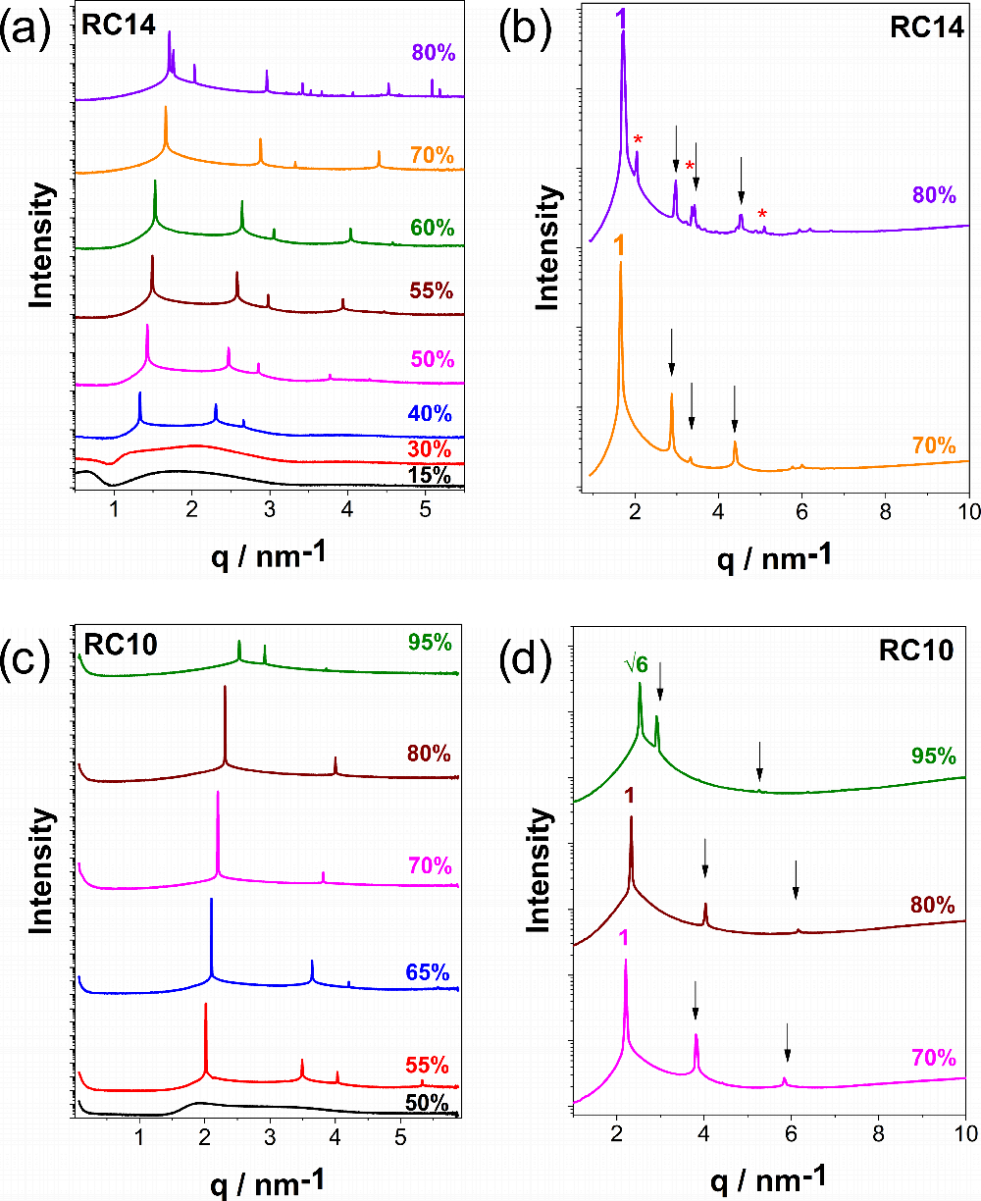
Dependence of scattering profiles on the concentration of RC14 (a, b) and RC10 (c, d) in water, as determined by SAXS/WAXS measurements. All measurements were performed at 25 °C. Arrows indicate the predicted sequence of mesophase peaks for a hexagonal phase (H_1_), except for RC10 at 95 wt %, where arrows indicate a bicontinuous cubic (Ia3d) mesophase. Asterisks denote peaks attributed to solid crystals, which are not correlated with the observed mesophase.

The X-ray scattering curves were used to identify the structures of the different aggregates. For RC14 at concentrations below 30 wt % (Figure 2a), the system exhibits a micellar (L_1_) phase, without the presence of Bragg peaks. As the concentration increases, a transition to an H_1_ mesophase is observed, occurring from approximately 40 wt % up to 70 wt %. The H_1_ mesophase was characterized by Bragg peaks positioned at ratios *q*_i_/*q*_1_ sequenced as 1:√3:√4:√7 (where “*i*” is the index for the reflection number). At 25 °C, above ca. 80 wt %, the system RC14 forms a solid crystalline phase, indicating strong molecular packing and reduced hydration.^32,33^ The H_1_-to-crystal transition occurs between 70 and 80 wt %, with the differences becoming more evident in the expanded view at these concentrations, as shown in Figure 2b. The H_1_ mesophase, in contrast to the L_1_ phase, displays optical birefringence, as observed with crossed polarizers (see Section 4 in the Supporting Information).

On the other hand, rhamnose C_10_ (RC10), as shown in Figure 2c, displays an extended region of micellar L_1_ phase, up to 50%, at higher concentrations than RC14, which is consistent with its shorter hydrophobic chain.^34^ RC10 forms a hexagonal (H_1_) mesophase within the concentration range of 55–80 wt %, as evidenced by the scattering profiles in Figure 2c and the characteristic optical birefringence between crossed polarizers (see Section 4 in the Supporting Information). At RC10 concentrations around 95 wt %, the formation of a bicontinuous cubic mesophase was detected and confirmed by the SAXS profile (see Section 5 in the Supporting Information). The Bragg peak positions for this cubic phase follow the √6:√8:√14 sequence, characteristics of the Ia3d space group. Notably, the Ia3d mesophase does not exhibit birefringence (see Section 4 in the Supporting Information). Bicontinuous (Ia3d) cubic phases were previously reported for glycolipids by Seddon et al. (1996).^35^ In contrast to this work, the Fd3m phase was identified in the vicinity of the reverse hexagonal (H_2_) and lamellar (L_α_) mesophases in xylose-based glycolipids.^35^ Interestingly, the formation of this Ia3d cubic mesophase was not observed in the RC14 system.

The values for lattice parameter (*a*) for the H_1_ formed by RC10 and RC14 were calculated (see Section 6 in the Supporting Information) and compared to further evaluate the effect of the alkyl chain length on the swelling behavior of the hexagonal phase,^33,36^ as represented in Figure 3.

**Figure 3:**
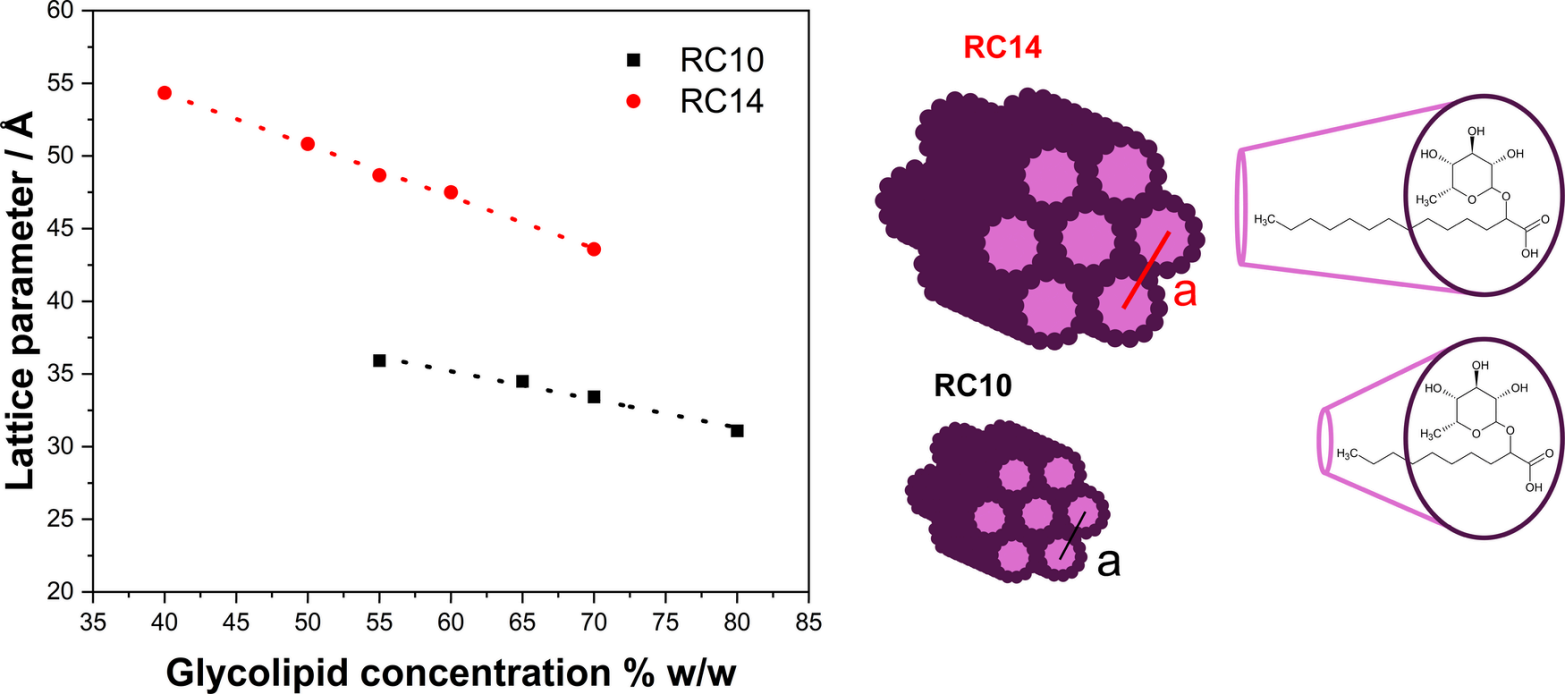
Dependence of the hexagonal phase (H_1_) lattice parameter (*a*) on glycolipid concentration for RC14 (black squares) and RC10 (red triangles). Measurements at 25 °C.

Both RC14 and RC10 exhibit a decrease in their hexagonal lattice parameter with increasing glycolipid concentration. However, this apparent linear behavior involves a very small change in lattice spacing, from 5.43 (40 wt %) to 4.34 nm (70 wt %) for RC14, and from 3.59 (55 wt %) to 3.11 nm (80 wt %) for RC10, and should not be considered universal for hexagonal phases, as reported in another study.^37^

Considering the well-known H_1_ approximations reported in the literature^38^ the internal radius for both glycolipids at different weight percentages can be compared, yielding radii of approximately 1.40–1.47 nm for RC10 and 1.80–1.90 nm for RC14 (see Section 6 in the Supporting Information). These values follow the same trend as those estimated for their micellar radii using the SAXS model, with values of 1.20 ± 0.01 and 1.57 ± 0.01 nm for RC10 and RC14, respectively. The slightly smaller values obtained from the SAXS micellar fitting are likely due to without considering the shell thickness. Nevertheless, all values follow the same trend, which may be attributed to the different methodologies used to estimate the radii in the L_1_ and H_1_ phases. This finding suggests that the aggregates grow predominantly in one direction upon the formation of hexagonal phases.

The slope of the straight boundaries in Figure 3, which indicates the swelling, is slightly larger for RC14 than for RC10, suggesting a less intense swelling of RC10. This could be associated with more water being incorporated into the cylinders of RC10 than RC14, therefore producing less separation among them as more water is added to the mixture, probably because of higher curvature radii for cylinders of the shorter RC10 glycolipid, making it possible to accommodate a larger fraction of water molecules at their interface.

### 3.4 Lyotropic Phase Behavior of Galactolipids GC10 and GC14

The structural characterization of glycolipids with galactose sugar headgroup, GC10 and GC14, at varying glycolipid concentrations (wt %) is represented by the SAXS curves shown in Figure 4.

**Figure 4:**
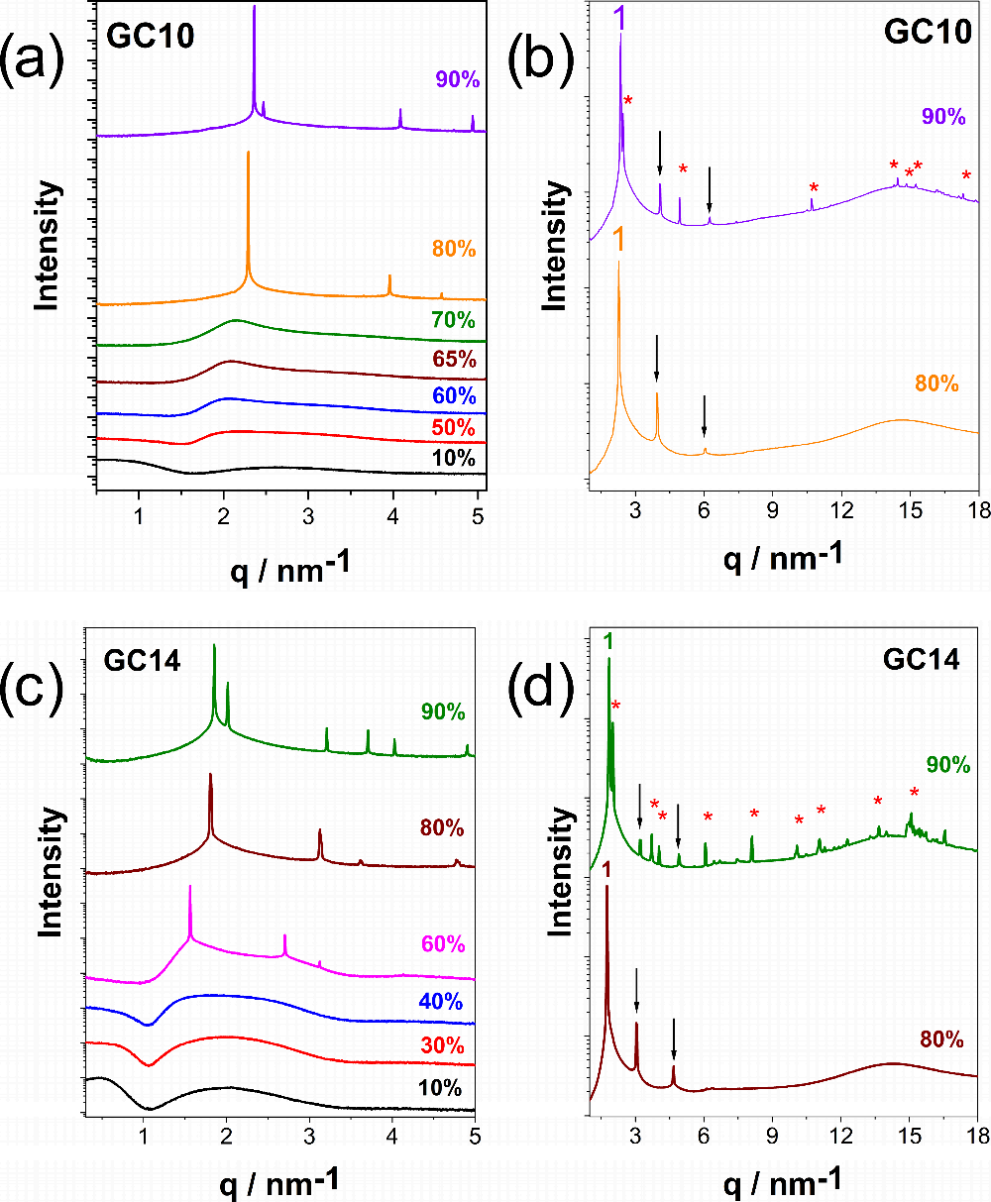
SAXS curves for various concentrations of aqueous galactolipids GC10 (a, b) and GC14 (c, d). Measurements at 25 °C for GC10 and 63 °C for GC14. Arrows indicate the mesophase sequence peaks expected for a hexagonal phase, and asterisks indicate solid crystals not correlated with the observed mesophase.

For GC10, at concentrations below 70 wt %, the system forms a micellar L_1_ phase, characterized by the absence of sharp Bragg peaks in their SAXS curves (Figure 4a). Upon increasing concentration, a transition to a hexagonal (H_1_) mesophase occurs between 70 and 80%, identified by the characteristic peaks positioned at ratios of 1:√3:√4 and the display of optical birefringence (see Section 4 in the Supporting Information). At 90 wt %, the system transitions into a solid crystalline phase, crossing its Krafft boundary, indicating enhanced molecular packing and significantly reduced hydration.^32,34^ The formation of this solid crystalline phase for GC10 is confirmed by the WAXS data (Figure 4b).

The SAXS curves representing the phase behavior of the longer chain galactolipid, GC14, are shown in Figure 4c. The GC14 lipid exhibits a shorter range of compositions for the micellar L_1_ phase than GC10, present only below 40 wt %, which is consistent with its higher hydrophobicity.^3^ GC14 transitions into an H_1_ mesophase at concentrations above 80 wt %, and to solid crystals at approximately 90 wt % (confirmed by WAXS, Figure 4d). Interestingly, GC14 exhibits the highest Krafft temperature (*T*_*k*_) among the four glycolipids investigated in this study, as previously noted (see Section 1 in the Supporting Information).

The unit cell parameters for GC10 and GC14 at 80 wt % were calculated using the diffraction peak positions (see Section 6 in the Supporting Information). From these values, the cylinder radii can be estimated as 1.49 nm for GC10 and 1.88 nm for GC14. These radii are consistent with the micellar aggregate dimensions discussed previously (see Table 1), confirming that the aggregates grow predominantly along one direction.

### 3.5 Comparing the Phase Behavior of Rhamno and Galactolipids

A summary of the binary phase boundaries determined for all the glycolipids is presented in Figure 5.

**Figure 5:**
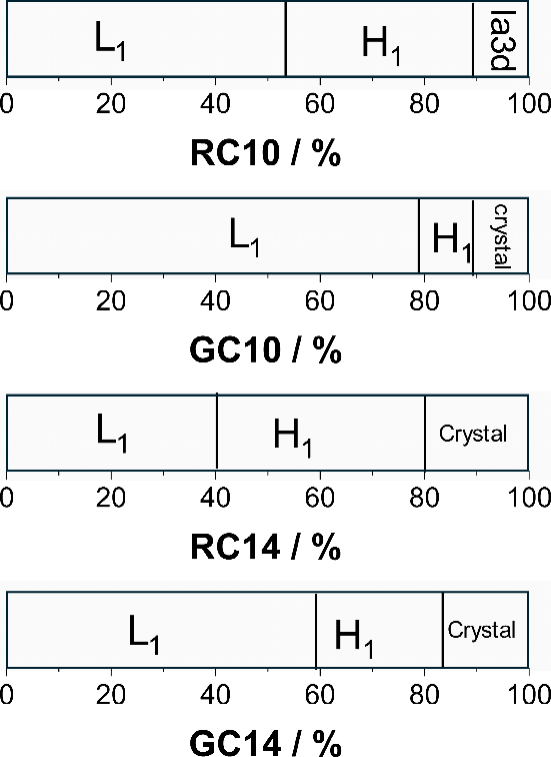
Binary phase boundaries determined for aqueous RC10 (25 °C), GC10 (25 °C), RC14 (25 °C), and GC14 (63 °C).

Comparison of the homologous pairs of rhamno- and galactolipids reveals a broader micellar phase region for the galactolipids. This indicates that the formation of high-curvature aggregates is more favorable in these systems, likely due to the distinct spatial arrangement of hydroxyl groups in the sugar headgroup, as previously^39^ and the same trend was observed in the micellization profile under buffer conditions.^20^ For this comparison, we assume that the degrees of ionization are the same due to similar p*K*_a_ values and, consequently, that the electrostatic interactions at their micellar interfaces are equivalent.

The H_1_ phases display different stability ranges (mostly due to the differences in micellar phases discussed above), but all appear to disappear around 90 wt %. When comparing glycolipids with the same sugar headgroup, H_1_ formation is directly influenced by the hydrophobic effect of the alkyl chain length. Furthermore, the concentration required for H_1_ formation is significantly lower for RC glycolipids than for GC glycolipids, which may be related to the higher water affinity of the galactose headgroup. In contrast, the termination of the H_1_ phase does not follow the same trend.

Interestingly, RC10 forms a bicontinuous cubic phase (Ia3d) above 95 wt % (a_Ia3d_ = 6.08 nm), which was not observed for GC10. This suggests a strong influence of the sugar headgroup on the packing behavior at high concentrations, unlike RC10, which did not crystallize within the investigated concentration range. On the other hand, GC10 may display distinct interactions with the solvent in the concentrated regime, where the presence of a small amount of water (less than 10 wt %) promotes crystallization.

This could indicate a preference for sugar–sugar rather than sugar–water interactions at higher concentrations. Such interactions may arise from the increased probability of hydroxyl groups forming hydrogen bonds when positioned in proximity, thereby favoring crystallization at lower concentrations than RC10. This behavior highlights not only the role of sugar hydrophilicity in lyotropic self-assembly, but also the importance of sugar–sugar headgroup interactions in the concentrated region.

In summary, the characterization of the bioinspired glycolipid micellar phases based on SAXS analyses confirmed the expected influence of alkyl chain length and sugar headgroup on micelle size, aggregation number, and micellar shape. These structural features directly correlate with their lyotropic phase behavior: longer chains favor H_1_ formation at lower concentrations, while sugar–water affinity and sugar–sugar interactions govern crystallization at higher glycolipid concentrations. The emergence of a cubic phase for RC10 and crystallization for GC10 highlight the distinct packing preferences imposed by their sugar headgroups. Overall, both hydrophobicity and headgroup interactions act to determine the phase boundaries of these ionic glycolipids.

## 4 Conclusions

This study elucidates the self-assembly of two families of bioinspired glycolipids in water. Overall, these results conform to the traditional sequence of phases reported for similar surfactants, showing the same trend when comparing glycolipids with different alkyl chain lengths. These micelles show no significant changes in aspect ratio, indicating that the micellar structure is not substantially altered upon concentration increase within the L_1_ region. The most interesting finding is that the formation of their hexagonal mesophases is noticeably more favorable for rhamno than galactolipids, indicating an important contribution from both hydration and sugar–sugar interactions for their self-assembly.

Collectively, these findings provide molecular-level insights into how subtle variations in the chemical structure of sugar headgroups, and hydrophobic chains govern their lyotropic phase behavior, with implications for designing glycolipid-based self-assembled systems for potential use in biotechnology and materials science. We believe that these findings, obtained with model glycolipids, are transferable to their more common commercial products, highlighting the relevance of this investigation.

## Supplementary Material



## Notes

Structural, experimental, and theoretical aspects of the study involving glycolipids; the determination of the Krafft temperature (*T_k_*) of GC14, a key parameter that defines the minimum temperature at which the glycolipid becomes sufficiently soluble to form micelles in aqueous solution, includes transmittance curves as a function of temperature, highlighting the transition between the crystalline and micellar states of the compound; the *G* function is introduced and defined within the context of the micelle fitting model; the estimated micellar parameters, obtained from fitting the theoretical model, includes equations for calculating micelle volume, aggregation number, and estimates of uncertainties by error propagation, as well as the structure factor value (*R*_HS_) derived from the SAXS fitting data; optical microscopy images of selected samples observed under crossed polarizers, revealing either birefringent or isotropic textures, depending on the sample, micrographs provide visual confirmation of phase formation and help identify the type of mesophase (such as hexagonal) formed, the optical observations complement the physicochemical data obtained from other techniques; the SAXS (small-angle X-ray scattering) curve of RC10 at 95 wt % is presented, confirming the presence of a bicontinuous cubic arrangement; the determination of the parameters of the hexagonal structure, based on the X-ray scattering results, parameters such as the lattice parameter of the hexagonal phase (*a*) and the intercylinder spacing (micelle radius), consolidates the structural interpretation, linking the experimental observations to the corresponding geometric model of GC14 organization; and the references that support the theoretical and experimental approaches, include sources related to experimental methodologies, micelle fitting models, and structural analysis techniques employed throughout the document (PDF)
