# Supplementary material for: Effect of the Chemical Structure of Ionic Glycolipids on Their Lyotropic Aqueous Phase Behavior
Source: ACS Omega. 2025 Nov 7;10(45):54929–38. doi: 10.1021/acsomega.5c08922 (PMC12631475; doi:10.1021/acsomega.5c08922)
Supplement: Supplementary file 1 [file ao5c08922_si_001.pdf]

Supplementary information

Effect of the chemical structure of ionic glycolipids on their lyotropic aqueous phase behavior

*Giuliana Valentini[a,b], Tomás S. Plivelic[c], Paulo R. A. F. Garcia[a], Shinji Kihara[b], Ben J. Boyd[b,d] and Watson Loh[a]**

[a]Institute of Chemistry, State University of Campinas (UNICAMP), P.O. Box 6154, 13083-970. Campinas, Brazil

[b]Department of Pharmacy, Faculty of Health and Medical Sciences, University of Copenhagen, Copenhagen 2100, Denmark

[c]MAX IV Laboratory, Lund University, 224 84 Lund, Sweden

[d]Drug Delivery, Disposition and Dynamics, Monash Institute of Pharmaceutical Sciences, Monash University, Parkville, VIC, Australia

Corresponding Author

* Corresponding author. E-mail: wloh@iqm.unicamp.br.

Present address for Giuliana Valentini: [a]Institute of Chemistry, State University of Campinas (UNICAMP), P.O. Box 6154, 13083-970. Campinas, Brazil.

Present address for Ben J. Boyd: [d]Drug Delivery, Disposition and Dynamics, Monash Institute of Pharmaceutical Sciences, Monash University, Parkville, VIC, Australia.

Table of Contents

Section 1. Krafft temperature of GC14……………….………………………………(4)

Section 2. Definition of the G function by the micelle fitting model……..………..…(5)

Section 3. Estimates of Micellar parameters ……………………….....…………..(6)

Section 4. Images of some samples under crossed polarizers ………..……………..…..(8)

Section 5. Small-Angle X-ray Scattering (SAXS)…………………………….………(9)

Section 6. Determination of parameters of the Hexagonal Structure ......…..……....(10)

Section 7. References……………………………………………………………...(12)

Section 1. Krafft temperature of GC14

1.1 Methodology

For GC14, 20 wt. % aqueous dispersions were prepared and gradually heated while monitoring optical transmittance at 600 nm. Suspensions containing crystalline surfactant scatter light strongly and therefore exhibit low transmittance. As the temperature rises and the crystalline phase dissolves, the solution becomes clear and the transmittance increases sharply. The Krafft temperature (T_K) was defined as the temperature at which the transmittance–temperature curve becomes essentially independent of further temperature increases. All measurements were performed in duplicate.

1.2 Results

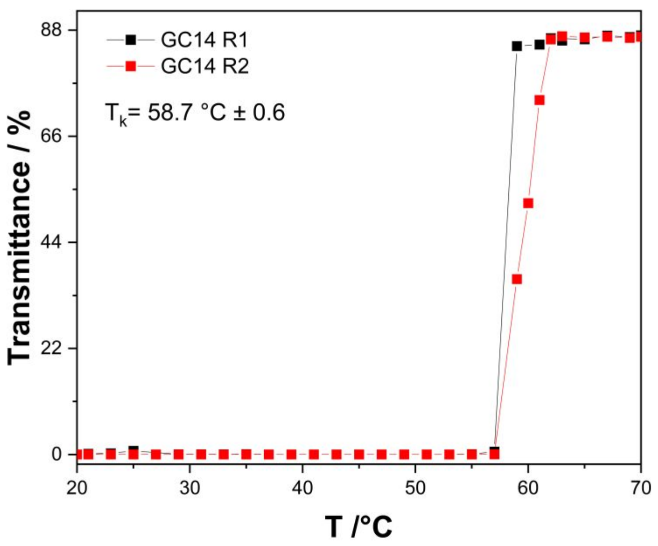

Figure S1. Krafft temperature of GC14 at 20wt.%. The GC14 experiment was conducted at a temperature range from 20°C to 70°C in duplicate. Above the Krafft temperature (T_k), the minimum temperature at which glycolipids form micelles in solution. Below this temperature, the glycolipid exists only as a hydrated crystal.

Section 2. Definition of the G function by the micelle fitting model

The G function describes oscillatory correlations arising from excluded-volume interactions among particles:

$$G(A) = \frac{\alpha\,[\sin(A) - A cos(A)]}{A^3} + \frac{\beta[2A\sin(A) + (2 - A^2)\; cos(A) - 2]}{A^4} + \frac{\gamma[-A^4\,\mathrm{cps}(A) + 4[(3A^2 - 6)\cos(A) + x^3 - 6A]\sin(A) + 6]}{A^5} \tag{S1}$$

With,

$$\propto = \frac{(1 + 2\eta)^2}{(1 - \eta)^4}$$

$$\beta = \frac{-\,6\eta(1 + \eta/2)^2}{(1 - \eta)^4} \tag{S2}$$

$$\gamma = \frac{\eta\alpha}{2}$$

The volume fraction (η) characterizes the hard-sphere model and an effective interaction radius (R_{HS}), which was approximated as the outer radius of the ellipsoids averaged over all orientations. The coefficients α, β, and γ depend on the volume fraction η.

Section 3. Estimates of Micellar parameters

3.1 Volume and aggregation number calculations

The length of the hydrocarbon chain (l_{chain}) was estimated using Equation S3, and the volume of the hydrophobic chain (V_{chain}) was calculated based on the Tanford model (Equation S4).[2,3] The ellipsoidal core volume is calculated using the fitting parameters, where the XY axes are a = b = R and the Z axis is defined as R × ϵ (Equation S5). For the core + shell volume, the shell thickness (t_h) is applied uniformly to all axes, and the calculation follows Equation S6. The aggregation numbers estimated without considering (Equation S7) and considering (Equation S8) the shell are described.

$$l_{chain} \ / nm = 0.154 + 0.1265\ (n) \qquad \text{S3}$$

$$V_{chain} \ / nm^3 = 27.4 + 26.9\ (n) \qquad \text{S4}$$

$$V_{core} \ / nm^3 = \frac{4}{3}\pi[R^2(\epsilon \cdot R)] \qquad \text{S5}$$

$$V_{core+shell} \ / nm^3 = \frac{4}{3}\pi[(R + t_h)^2 \cdot [(\epsilon \cdot R) + t_h] \qquad \text{S6}$$

$$N_{agg\ core} = \frac{V_{core}}{V_{chain}} \qquad \text{S7}$$

$$N_{agg\ core+shell} = \frac{V_{core+shell}}{V_{chain}} \qquad \text{S8}$$

3.2. Estimates of uncertainties by error propagation

The uncertainties in derived parameters such as the core and core–shell micelle volumes, as well as the apparent aggregation number, were estimated by standard propagation of uncertainty from the fitted parameters R, t_h, and ϵ. The analytical approach follows the general law of error propagation [2], assuming uncorrelated errors in the primary parameters by Equation S9:

$$(\Delta f)^2 = \sum_i \left(\frac{\partial f}{\partial x_i}\right)^2 (\Delta x_i)^2 \qquad \text{S9}$$

Where Δf is the derived quantity, x_i is the fitted parameter, and Δx_i is their uncertainty. For the previously described core ellipsoid volume, the relative uncertainty was calculated using Equation S10:

$$\frac{\Delta V_{core}}{V_{core}} = \sqrt{9(\Delta R/R)^2 + (\Delta\epsilon/\epsilon)^2} \qquad \text{S10}$$

Considering the shell, the relative uncertainty was calculated using Equation S11:

$$\frac{\Delta V_{core+shell}}{V_{core+shell}} = \sqrt{(\Delta R)^2 + (\Delta t_h)^2} \qquad \text{S11}$$

Finally, the uncertainty in the apparent aggregation number, defined as the ratio between micelle volume and the molecular chain volume (assumed known without uncertainty), was obtained as Equations S12 and S13, respectively:

$$\Delta N_{app\ core} = \frac{\Delta V_{core}}{V_{tail}} \quad \text{S12}$$

$$\Delta N_{app\ core+shell} = \frac{\Delta V_{core+shell}}{V_{tail}} \quad \text{S13}$$

3.3 Structure factor

Table S1. R_{HS} is the parameter related to the structure factor, representing the interaction distance for a spherical approximation.

	**RC10 50%**	**RC14 30%**	**GC10 70%**	**GC14 40%**
**R_{HS} / nm**	1.762 ±0.002	2.827 ±0.003	1.591 ±0.001	2.209 ±0.005

Section 4. Images of some samples under crossed polarizers

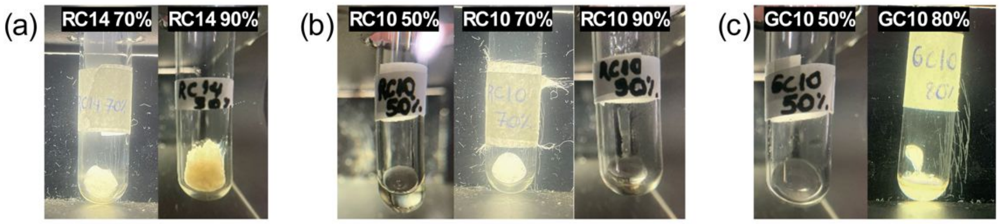

Figure S2. Photography of the samples taken between crossed polarizers: (A) RC14 samples: 70 wt.% hexagonal mesophase (a) and 90 wt.% solid crystals (b); (B) RC10 samples: 50 wt.% micellar phase (a), 70 wt.% hexagonal mesophase (b), and 90 wt.% bicontinuous cubic phases (c); (C) GC10 50 wt.%% micellar phase (a), 80 wt.% hexagonal mesophase (b).

Section 5. Small-Angle X-ray Scattering (SAXS)

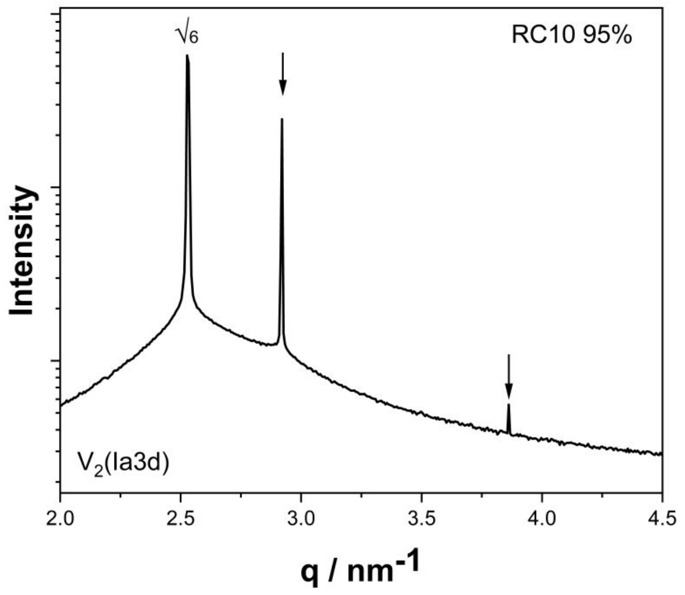

Figure S3. SAXS curve for the bicontinuous cubic phase of RC10 at 95 wt.%. Arrows indicate the mesophase sequence peaks expected for a cubic Ia3d mesophase. ($q_{\sqrt{6}}$= 2.5296 nm^{-1})

Section 6. Determination of parameters of the Hexagonal Structure

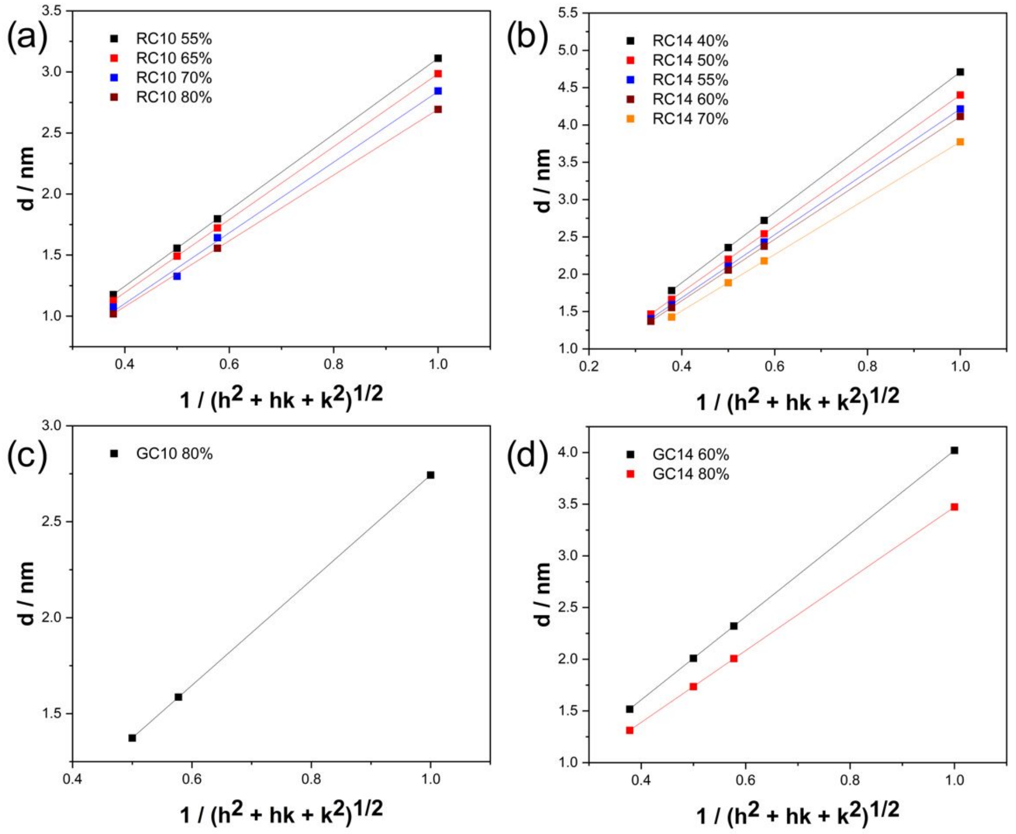

Figure S4. A linear fit, intersecting the origin, is obtained from a plot of the *d*-values of the observed peaks versus $1/\sqrt{(h^2 + hk + k^2)}$ for space group H_1 for RC10 (a), RC14 (b), GC10 (c), and GC14 (d).

Table S2. Hexagonal lattice parameters for various concentrations of RC10, RC14, GC10, and GC14.

Glycolipid / wt. %	Lattice parameter / nm			
	RC10	RC14	GC10	GC14
40	-	5.43	-	-
50	-	5.08	-	-
55	3.59	4.87	-	-
60	-	4.75	-	4.64
65	3.45	-	-	-
70	3.34	4.34	-	-
80	3.11	-	3.16	4.01

Values determined at 25 °C for RC10, RC14, GC10 and 63 °C for GC14.

The cylinder radius R of the H_1 mesophase is correlated with the lattice parameter (a) and the volumetric surfactant fraction (ϕ_{surf}) as previously described [1] by:

$$R\ /\ nm = \sqrt{\frac{\sqrt{3}\, a^2 \phi_{surfactant}}{2\pi}} \qquad \text{(S14)}$$

Considering the solvent density is the same as the glycolipids (approximated as 1.0 g cm^{-3}), the $\phi_{surfactant}$ was calculated by:

$$\phi_{surf} = \phi_{total} - \phi_{water} \quad \text{(S15)}$$

Table S3. Micelle radius for various concentrations of RC10, RC14, GC10, and GC14.

Glycolipid / wt. %	Micelle radius / nm			
	RC10	RC14	GC10	GC14
40	-	1.80	-	-
50	-	1.88	-	-
55	1.40	1.89	-	-
60	-	1.90	-	1.88
65	1.46	-	-	-
70	1.47	1.90	-	-
80	1.46	-	1.49	1.88

Values without parentheses represent the glycolipids weight percentage, while the lattice parameter is given in parentheses (a).

Section 7. References

[1] Asghar KA, Rowlands DA, Elliott JM, Squires AM. Predicting Sizes of Hexagonal and Gyroid Metal Nanostructures from Liquid Crystal Templating. *ACS Nano* **2015**, *9*, 10970–8. https://doi.org/10.1021/acsnano.5b04176.

[2] Tanford C. The Hydrophobic Effect: Formation of Micelles and Biological Membranes. 2nd Edition. *Wiley*, **1980**.

[3] Israelachvili JN. Soft and Biological Structures. In Intermolecular and Surface Forces. 3rd Edition. *Elsevier*, **2011**, p. 535–76.
